# Design and Testing of MEMS Component for Electromagnetic Pulse Protection

**DOI:** 10.3390/s25010221

**Published:** 2025-01-02

**Authors:** Shiyi Li, Hengzhen Feng, Wenzhong Lou, Yuecen Zhao, Sining Lv, Wenxing Kan

**Affiliations:** 1 School of Mechatronical Engineering, Beijing Institute of Technology, Beijing 100081, China; 2 Chongqing Innovation Center, Beijing Institute of Technology, Chongqing 401120, China; 3 Science and Technology on Electromechanical Dynamic Control Laboratory, Beijing 100081, China; 4 Department of Micro/Nano Electronics, School of Electronic Information and Electrical Engineering, Shanghai Jiao Tong University, Shanghai 200240, China

**Keywords:** strong electromagnetic environment, electromagnetic energy diversion, response characterization, safety protection, MEMS

## Abstract

With the demand for high-safety, high-integration, and lightweight micro- and nano-electronic components, an MEMS electromagnetic energy-releasing component was innovatively designed based on the corona discharge theory. The device subverted the traditional device-level protection method for electromagnetic energy, realizing the innovation of adding a complex circuit system to the integrated chip through micro-nanometer processing technology and enhancing the chip’s size from the centimeter level to the micron level. In this paper, the working performance of the MEMS electromagnetic energy-releasing component was verified through a combination of a simulation, a static experiment, and a dynamic test, and a characterization test of the tested MEMS electromagnetic energy-releasing component was carried out to thoroughly analyze the effect of the MEMS electromagnetic energy-releasing component. The results showed that after the strong electromagnetic pulse injection, the pulse breakdown voltage of the MEMS electromagnetic energy-releasing component increased exponentially in terms of the pulse injection voltage, and the residual pulse current decreased significantly from one-third to one-half of the original, representing a significant protective effect. In a DC environment, the breakdown voltage of the needle–needle structure of the MEMS electromagnetic energy-releasing component was 144 V, and the on-time was about 0.5 ms.

## 1. Introduction

The safety enhancement of a sensitive electronic component under a high-power electromagnetic pulse (EMP) excitation is a key, core technology in circuit safety [1,2,3]. The peak field strength of a high-power EMP can reach tens of thousands of V/m, and the peak power can reach as high as the GW level, which can easily cause circuit failure and seriously threaten the safety of electronic systems [4,5,6]. Therefore, the electromagnetic protection of sensitive electronic components has been a key research area.

Research has focused on system-level, device-level, and component-level protection [7,8] from three perspectives: theoretical calculations [9,10,11], numerical simulations [12,13,14], and simulation tests [15,16]. Zhang et al. [17,18,19] adopted a ferrite material to sinter ferrite inside fireworks or connect ferrite beads in series in foot wire. It was used to absorb and attenuate high-frequency-induced currents in electrical fireworks. Typical pyrotechnic products and low-firing-energy pyrotechnic products do not fire in radio frequency (RF) experiments at a power of 20 W. Ferrite beads can effectively absorb and attenuate high-frequency signals and electrostatic interference. Tony L. King et al. [20,21] connected capacitors in parallel at both ends of the pyrotechnic device. They utilized the low-impedance effect of capacitance in a high-RF environment to realize the electromagnetic protection of sensitive electronic devices. This method leaves the firing threshold voltage of the pyrotechnic unaffected. The results show that a 1μF capacitor provides the best protection for the pyrotechnic device against the hazards of electrostatic discharge. A 14 V Zener diode was also connected in parallel at both ends of the artifact. The electrostatic protection of the pyrotechnics was realized. Ren et al. [22,23] utilized the transient voltage suppression function of the transient suppression diode of the RF protection of pyrotechnics-related research. After the protection of the pyrotechnic products, the electro-explosive performance had no effect. It could effectively inhibit the interference of radio frequency in pyrotechnic products. Ye et al. [24,25] implemented a parallel thermistor at both ends of the pyrotechnic products. They utilized the junction capacitance of the varistor. Junction capacitance has a certain electromagnetic protection ability. This meant that the thermistor combination of low-pyrotechnic products in the electrostatic discharge experiment did not fire. This effectively improved the anti-electromagnetic capability of the pyrotechnic products. Du et al. [26,27,28,29] proposed the electromagnetic protection of pyrotechnics using an integrated protection circuit. In constant-current excitation conditions, the critical non-fire current of pyrotechnic products was increased from 0.9 A before protection to 1.4 A. This greatly improved the constant-current safety performance of the pyrotechnic products. Xue et al. [30,31,32] used additional matched filters to design electromagnetic protection for semiconductor bridges. This method attenuates high-frequency electromagnetic energy by a factor of 1000. It has a significant attenuation effect on the energy coupled in the high-frequency electromagnetic field. Table 1 shows the advantages and disadvantages of various traditional protection methods.

These studies provide much support and many references for this paper. Still, in the face of a high-power electromagnetic pulse, a new conceptual surface killing element, these protection means are based on traditional components for pulse protection. Traditional protection circuits and devices are large and cannot be assembled in large quantities. There is an urgent need for a small size, strong anti-interference ability, and good protective attenuation effect of micro-small electromagnetic energy-releasing components for sensitive parts of the electromagnetic protection. In this paper, an MEMS electromagnetic energy-releasing component (MERC) is designed based on the principle of micro-corona discharge and MEMS process, and the electromagnetic energy-releasing ability of MERC is tested through a combination of simulation, static test, and dynamic experiment, to ensure that it can effectively protect against accidental firing of pyrotechnic products.

## 2. Design

### 2.1. Design of Evacuation Component Based on Corona Discharge

**Figure 1:**
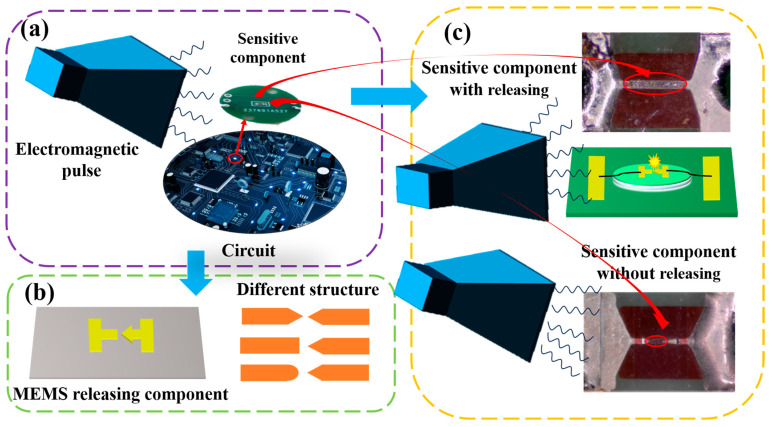
MERC working principle. (**a**) Schematic diagram of a sensitive component to the effects of the electromagnetic environment. (**b**) Schematic diagram of the structure of the MERC. (**c**) Comparison of the effect of the sensitive component with and without the MERC.

As shown in Figure 1a, due to the electromagnetic environment, there is a need to design a micro-system with high anti-interference capability and a small size for electromagnetic energy channeling. As shown in Figure 1b, a MERC based on corona discharge is proposed in this paper. It can be processed to produce a micro gas gap at the micrometer scale by MEMS processing. It includes two pads and two electrodes for gas breakdown discharge. It utilizes a conductive path formed by the breakdown of the gas gap at high voltage. It generates the main energy channel that conducts low-energy-sensitive devices. This method can effectively channel the flow of electromagnetic energy through the sensitive device. Due to the inter-electrode gap being at the micron level, it has a fast conduction speed and is voltage-sensitive. This component greatly enhances the safety of the protected device.

As shown in Figure 1c, the MERC is connected in parallel to both ends of the protected device. When no abnormal electromagnetic energy passes through, due to the air gap between the two electrodes of the MERC, it is in a circuit breaker state. When the abnormal electromagnetic energy passes through the releasing component, an uneven electric field is generated between the two electrodes. The gas between the two-electrode gap begins to release. When the abnormal electromagnetic energy increases to the point that the field strength between the poles exceeds the insulation degree, the gap between the two poles will be discharged to break down. The gap from the original insulating state transforms into a conductive state. At this time, the releasing component between the plasma formation establishes a small resistance conductive channel. It constitutes an electromagnetic energy discharge channel so that much energy is discharged. Thus, the protected device can be safe.

### 2.2. Gas Breakdown Simulation

**Figure 2:**
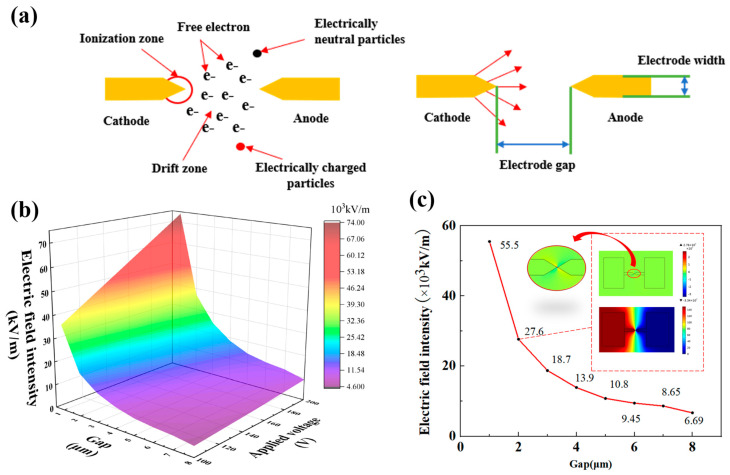
Principle and simulation analysis. (**a**) Electrode breakdown principle based on corona discharge. (**b**) Electrode breakdown field strengths under different electrode gap sizes and applied voltages. (**c**) Electrode breakdown field strengths under different electrode gaps at an applied voltage of 150 V.

The MERC occurs when gas breakdown is the essence of corona discharge. The gas gap breakdown of the field strength size is related to the electromagnetic releasing component and can form an effective protection of sensitive components of the key. Figure 2a is the structure of the miniature corona ion excitation and the corresponding discharge process. Therefore, safety protection can be better realized through reasonable design. According to the work of a large number of researchers, it is known that the structural factors affecting the electrode corona discharge mainly include the electrode shape and electrode gap. The empirical formula is given in [33,34].

(1) $$E=\frac{2V}{rln\left[\left(\frac{2d}{r}\right)+1\right]}$$

where E is the electric field strength of the distortion during electrode breakdown, r is the radius of curvature of the tip where surface ionization occurs, d is the electrode gap size, and V is the applied voltage at the ends of the electrode.

By analyzing Formula (1), it can be seen that the field strength at electrode breakdown is proportional to the gap of the electrode and inversely proportional to the curvature of the electrode tip. The precision of the electrode gap size is greatly affected by the MEMS component process, which in turn affects the breakdown threshold of the gas gap. Therefore, this paper first analyzes the breakdown thresholds of electromagnetic releasing components with different gap sizes. Through COMSOL simulation software, the MERC with different electrode gaps was applied at 100 V, 150 V, and 200 V for simulation. This method mainly determines whether the MERC can reach the air breakdown field strength (30 kV/cm).

The needle–needle electrode is selected as an example for simulation calculation. To explore the relationship between the electrode inter-electrode electric field strength and the size of the electrode gap at the time of breakdown, the simulation parameters are shown in Table 2.

As shown in Figure 2b, the size of the electrode gap affects the field strength during electrode breakdown discharge. When the electrode gap is constant, the electric field strength is gradually enhanced with the enhancement of the applied voltage. When the applied voltage is constant, the electric field strength is gradually weakened with the increase in the electrode gap, which is by the law of Formula (1). As shown in Figure 2c, applying a constant voltage of 150 V to the MERC with different gaps, the size of the field strength between the electrodes decreases gradually with the increase in the gap. Due to the actual processing accuracy limitation, the smaller the gap, the higher the processing cost. Although the breakdown field strength of a 1 μm electrode gap is significantly higher than that of a 2 μm electrode gap, the cost will be significantly higher for a gap smaller than 2 μm to be lithographed step by step to ensure the lithography’s precision. Therefore, while minimizing the electrode gap as much as possible and taking into account the actual processing cost, 2 μm is selected as the electrode gap of the MERC.

## 3. Experiment

### 3.1. Process

In order to realize high-precision and low-cost MERC processing, this paper adopts the MEMS thick film surface silicon process and selects gold as the electrode of MERC to produce MERC chips.

**Figure 3:**
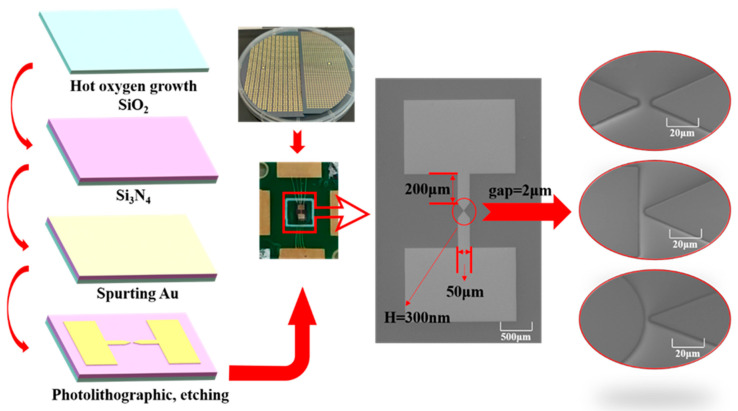
Processing flow charts and objects.

The processing flow is shown in Figure 3. Firstly, 500 nm silicon dioxide is grown on the surface of the substrate single-crystal silicon wafers by a thermal oxidation process. Then, a layer of 500 nm silicon nitride is grown by LPCVD to ensure insulation and prevent the structure from constituting an internal short circuit. After that, a layer of 10 nm chromium is sputtered as an adhesion layer to increase the adhesion of the electrode material to the substrate. Finally, a layer of 100 nm gold is sputtered and etched to form the electrodes of the MERC. The processed structural metal lines are uniform in width and the electrodes are shaped. The electrode shape is another significant influence on gas breakdown. To obtain the influence law of different electrode shapes on gas breakdown, three different electrode shapes are processed in this paper.

### 3.2. Tests on the Protection Ability of Different Electrode Shapes

**Figure 4:**
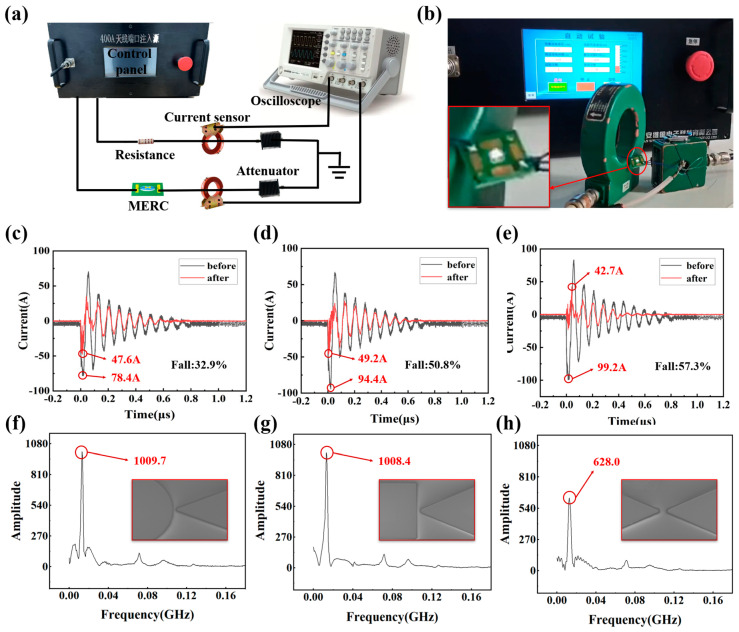
Test and test results. (**a**) Schematic diagram of test equipment and test circuit for protection ability test. (**b**) Test effect. (**c**) Time-domain curve of residual current after injecting current in needle−spherical structure. (**d**) Time-domain curve of residual current after injecting current in needle−planar structure. (**e**) Time-domain curve of residual current after injecting current in needle−needle structure. (**f**) Frequency-domain curve of residual current in needle−spherical structure. (**g**) Frequency-domain curve of residual current in needle−planar structure. (**h**) Frequency-domain curve of residual current of needle−needle structure.

In order to analyze the influence of different electrode shapes on the gas breakdown threshold, this paper uses the HEMP-PCI pulsed impulse current injection source to test the MERC. Test the protection performance of MERC with different electrode shapes. The test schematic diagram is shown in Figure 4a. The test device consists of a pulse generator, load resistor, oscilloscope, pulse current probe, attenuator, and electromagnetic shielding box. The MERC to be tested is connected to both ends of the pulse generator. At the same time, the pulse current probe is connected to the circuit where the MERC and the load resistor are located and then connected to the oscilloscope through the connecting wire.

Different electrode shapes of MERC are tested. The response waveforms were obtained by injecting a pulse current of 100 A. When the MERC reaches the pulse breakdown voltage. The MERC conducts, and the voltage at both ends decreases rapidly. It finally enters into the state of arc-light discharge and stabilizes the voltage in a very small range.

As can be seen from Figure 4c–e, the residual pulse current through the MERC with different electrode shapes is significantly reduced to one-third to one-half of the original one. In particular, the over-pulse current can be effectively suppressed at the first peak moment of the strong pulse coming over, limiting it to a low level. Among them, the needle–needle electrode can attenuate the initial 99.2 A current to 42.7 A, which is a 57.3% attenuation. The protection effect is the most obvious. The residual pulse current curve was transformed into a residual voltage curve, which is then converted into a waveform spectrogram by Fourier transform. The spectrogram shows that the voltage frequency is relatively rich in components below 500 MHz, and the frequency peak is more concentrated at 120–130 MHz. The frequency peak at 129.98 MHz for the needle–planar structure is 1008.4, the frequency peak at 129.98 MHz for the needle–spherical structure is 1009.7, and the frequency peak at 124.98 MHz for the needle–needle structure is 628.9. The amplitude of all other frequencies is relatively low, which controls the perturbation at a low level. Therefore, the needle–needle electrodes have better evacuation performance in the case of limitations due to the machining process.

### 3.3. DC Breakdown Threshold Test

**Figure 5:**
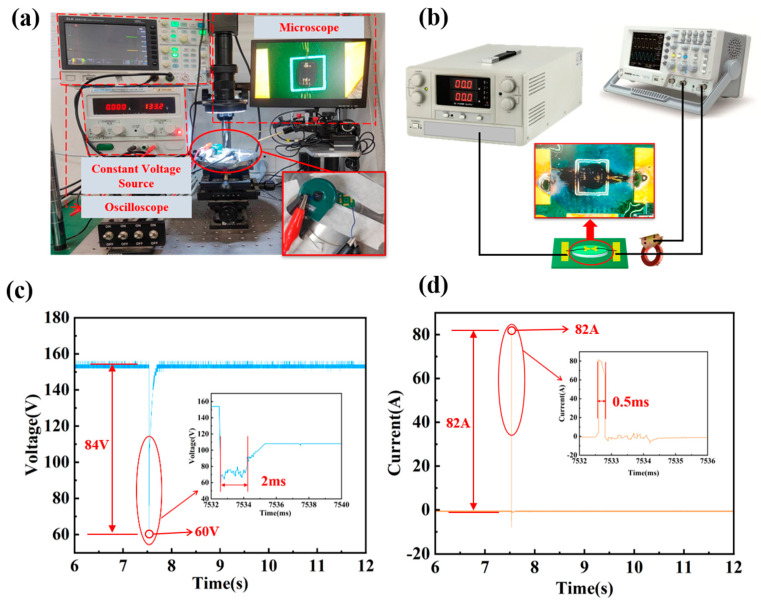
Test and test results. (**a**) DC breakdown threshold test equipment. (**b**) DC breakdown threshold test circuit schematic diagram. (**c**) DC breakdown voltage over time curve. (**d**) DC breakdown current over time curve.

In order to test the breakdown parameters of the MERC, the DC breakdown threshold test of the MERC is carried out. The DC breakdown threshold test equipment and schematic diagrams are shown in Figure 5a,b. The DC breakdown threshold test system mainly includes a voltage generator, an oscilloscope, a voltage probe, a current probe, a microscope, and a multimeter. The voltage generator is used to provide pulse voltage to the MERC. The oscilloscope, voltage probe, and current probe are used to measure the voltage and current signals of the MERC. The microscope is mainly used to observe the phenomena before and after the action of the MERC. The multimeter is used to check whether the performance of the switch is intact before testing.

The test was conducted in an environment with a temperature of about 25 °C and a humidity of about 30%. The voltage is applied to the MERC by the step method. The voltage is gradually increased until breakdown occurs. The DC breakdown voltage damage threshold provides a database for the effect of an electromagnetic pulse on electronic circuits. 

When a breakdown occurs, a strong light appears, accompanied by an explosion sound, and the electrode metal fuses. As shown in Figure 5c,d, the DC breakdown voltage drops abruptly by 84 V and then reaches 60 V. This is a process of channel conduction. After the end of conduction, the DC voltage rises gradually. The current rises from 0 A to 82 A. After the end of conduction, the current falls. This is because during the discharge process, a high-temperature arc is generated internally, which consumes the electrons on the electrode surface and generates plasma. To realize the breakdown discharge, more energy is needed to generate the initial electrons, which also causes the voltage to change, and this process is about 2 ms. The discharge ends and the voltage is restored. The on-time current changes at the same time as the voltage changes. This process includes the time required for the plasma to continuously diffuse through the inside of the electrode gap to make the main circuit act normally, about 0.5 ms. This time is also the on-time of the DC voltage breakdown discharge of the MERC.

### 3.4. Protective Capability Test Under Different Pulse Injection Currents

**Figure 6:**
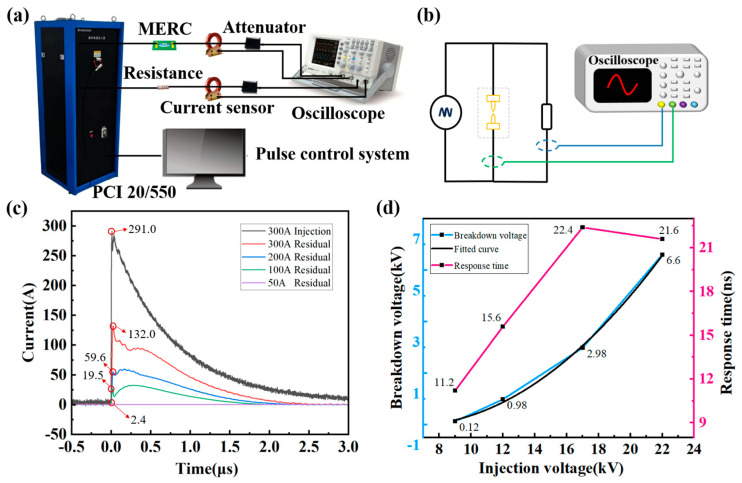
Test and test results. (**a**) Protective capability test equipment. (**b**) Protective capability test circuit schematic. (**c**) Residual current time-domain curves after different sizes of injection currents. (**d**) Breakdown voltage and response time curves with the variation of injection voltage.

To verify the protection ability of MERC under different sizes of injected current, this paper employs the IEC61000-4-24 standard; the test adopts the PCI 20/550 type electromagnetic pulse impulse current simulation system, whose standard waveform is a double-exponential impulse current wave of 20/550 ns, and the source impedance is 60 Ω, and the injected test is carried out on MERC. The PCI pulsed current injection test equipment and principle are shown in Figure 6a,b. The injection test system consists of a signal generation system, evacuation load system, and data acquisition system. The signal generating system is mainly composed of a PCI20/550 type EMP pulse current simulation system, which outputs the EMP signal to meet the test conditions and injects it into the evacuation load system. The releasing system is mainly composed of MERC and load impedance. The dynamic test verifies the attenuation effect of electromagnetic pulse protection of MERC. The data acquisition system is mainly composed of an oscilloscope, pulse current probe, attenuator, and electromagnetic shielding box, to collect the pulse current waveform of the MERC in the process.

The designed MERC is connected in parallel with the 2 Ω load impedance into the measurement loop. The oscilloscope CH1 port measures the pulse current injection waveform of the pulsed high-voltage source through the pulse current sensor. The CH_2_ port measures the residual pulse current of the MERC through the pulse current sensor. Different levels of pulse currents of 50 A, 100 A, 200 A, and 300 A are injected into the MERC, respectively. The collected residual current waveforms are shown in Figure 6c.

From Figure 6c, it is found that the change in the residual pulse current after the MERC is obvious. The whole waveform is obviously reduced by two to three times compared with the injected pulse current waveform, with an obvious protective effect.

Table 3 shows the specific values of the test residual pulse current. The pulse current rejection ratio in the table is calculated by the following formula:

(2) $$SE_{v}=20\mathrm{lg}\frac{I_{p}}{I_{r}}$$

where $SE_{v}$ is the pulse current rejection ratio, $I_{p}$ is the peak pulse current injection, and $I_{r}$ is the peak residual pulse current.

As can be seen from Table 3, the pulse current rejection ratio is 10.3 dB at the injected pulse currents of the order of 100 A and 200 A. The protection effect is relatively obvious.

The pulse amplitude is expressed in terms of voltage. The injected pulse current amplitudes of 50 A, 100 A, 200 A, and 300 A are converted into voltages of about 9 kV, 12 kV, 17 kV, and 22 kV. The response waveforms are captured by using an oscilloscope. The data are extracted and converted into voltage waveforms. Scatter plots of injection voltage versus pulse breakdown voltage are plotted and fitted according to the test results. It can be seen from Figure 6d that the pulse breakdown voltage of the MERC has an exponentially increasing relationship with the pulse injection voltage. Formula (3) is fitted according to the measured data.

(3) $$U_{s}=0.7425\exp\left(\frac{U}{9.0429}\right)-1.8630\;R^{2}=0.9998$$

where $U_{s}$ is the pulse breakdown voltage and U is the peak injection voltage, and the fitting factor $R^{2}$ is more than 0.9.

In Figure 6d, it can be seen that the response time of the MERC is roughly between 10 and 25 ns, which indicates that the MERC can respond in time to the fast-pulse voltage. It has a good role in draining the steeper ns-level pulse voltage. With the increase in the injected voltage, the response time of the MERC shows a tendency to increase first and then decrease. This variation in response time may be related to the air breakdown characteristics. When a certain applied voltage height is reached, the gas ionization is faster, and the initial electrons that can be generated faster collide with the gas molecules and conduct quickly.

## 4. Validate

### 4.1. Surface Morphology Analysis

**Figure 7:**
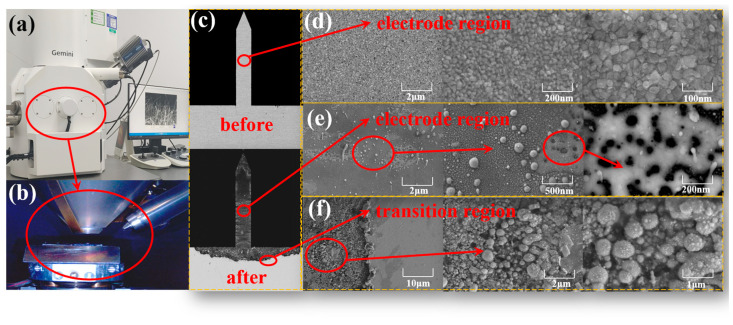
Validate results. (**a**) Field Emission Scanning Electron Microscope (FESEM). (**b**) Photographic process. (**c**) Electrode surface topography before and after the test. (**d**) Electrode surface without pulsed current applied. (**e**) Electrode surface with pulsed current applied. (**f**) Transition region of the electrode surface with pulsed current applied.

To further explore the physical component, the surface morphology of the post-test electrode region and the post-test reacted and unreacted transition region after the 100 A pulse current impact is analyzed. The field emission scanning electron microscope is used to take pictures, as shown in Figure 7a,b, and the surface morphology in different states is shown in Figure 7c. The regions of Figure 7d are the uniform regions in the middle of the electrode when the 100 A pulse current is not applied. The original surface morphology is uniform and grain-like, with the presence of micro protrusions, which can form discharge channels. Figure 7e shows the electrode area under the action of 100 A pulse current; the surface morphology of the electrode has changed significantly, which is mainly manifested in the formation of a molten pool and the formation of nanoscale spherical particles. The surface electrode is eroded and there is a significant warping appearance. The surface precipitates nanoscale spherical particles of different sizes. There is some agglomeration between the particles, accompanied by the formation of a melt pool. With the growth of the action time, the erosion pool tends to gradually expand, and gradually form a “full-scale electric explosion”. The morphology of the material in the electrode area changes. With the growth of the energization time, the electrode material vaporization is blown away by the blast wave, while the subsequent strong ionization will be produced, accompanied by strong plasma radiation. Due to the thin electrodes and less surface residue, most of the sputtered particles are stacked to form ablation blocks of various shapes under the action of electric field force. With the increase in the number of discharges, the electrode material is continuously consumed, leaving only a few spherical particles and dispersed molten pools, making it difficult to form subsequent discharge channels. Figure 7f shows the surface morphology of the transition region between the electrode and pad after the action of a 100 A pulse current. It can be seen that there are more spherical particles. In conclusion, the surface morphology after discharge of different forms varies greatly, which may be related to the structure of the material itself and the manufacturing process. In terms of discharge life, the “full-scale electric explosion” will consume a large amount of electrode material, and the MEMS chip itself has a tiny thickness, resulting in a relatively short discharge life of the component.

### 4.2. Elemental Analysis

**Figure 8:**
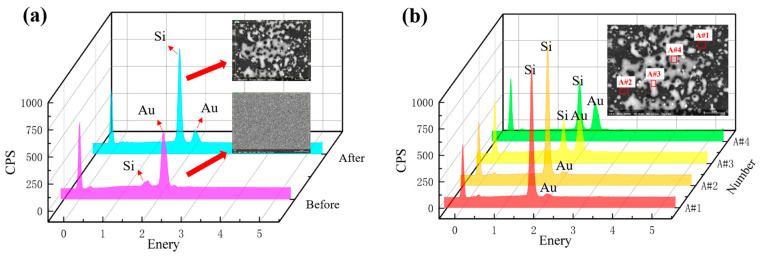
Validate results. (**a**) Elemental analysis of the electrode surface before and after the test. (**b**) Elemental analysis of different areas of the electrode surface after the test.

To further confirm the consistency of the electrode changes with those observed in the surface morphology, elemental analysis is performed on the electrode area before and after the test. The results of the analysis are shown in Figure 8.

As can be seen from Table 4 and Figure 8a, the initial electrode surface is mainly gold elements, with a small amount of silicon elements in the substrate and carbon elements in the conductive adhesive in the EDS sampling process. The atomic mass of the gold element is 94.14%, which can ensure the purity of the electrode and promote the stable discharge of the electrode. The content of carbon and silicon is relatively low, with an atomic mass of 3.16% and 2.71%, which has a negligible effect on the electrode discharge.

After discharge, the electrode surface is severely depleted, with the atomic masses of the gold and silicon elements being 45.80% and 35.86%, respectively. The gold element on the electrode surface is gradually consumed. It results in the electrode surface being dominated by the silicon element of the substrate, accompanied by a small amount of chromium element in the adhesive layer and nitrogen element in the insulating layer (silicon nitride). In the discharge process, the gold electrode undergoes gasification and ionization during sputtering. Some are blown away. Others are sputtered to other positions of the electrode to form a molten pool. It cools and forms ablative blocks. Large areas of the substrate are exposed. There is elevated silica content.

Table 5 and Figure 8b show the energy spectrum analysis of four small regions after the selection of the test. Regions A#1 and A#2 have serious substrate exposure, which is because the gold electrode in this region is sputtered relatively completely by gasification and ionization in the process of discharge, and there is less residue. Regions A#1 and A#2 are mainly the formation of ablated blocks, which are mainly formed from the sputtered particles of this and the surrounding regions under the effect of gravity after cooling. This region is mainly for the sputtered particles in this region and the surrounding region formed by gravity after cooling, with a small number of ablation craters.

Combined with the electrode ablation morphology, elemental changes can be seen in the discharge breakdown. The structure and morphology of the entire electrode changed from a dense, uniform electrode into a sparse, uneven electrode consisting of numerous ablative blocks. The electrode gap increases significantly and the performance changes. It is less susceptible to subsequent breakdown.

### 4.3. Verification of the Protective Properties of Pyrotechnic Products

**Figure 9:**
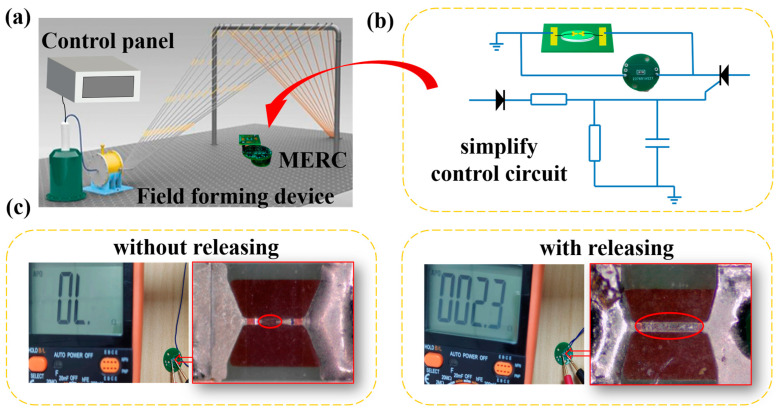
Validate results. (**a**) Sensitive electronic component protection performance verification test equipment. (**b**) Sensitive electronic component protection performance verification test circuit schematic. (**c**) With or without MERC sensitive electronic component role effect.

The EMP irradiation source is used to test the MERC and pyrotechnic products. It can test the state of pyrotechnic products with or without the MERC. The test schematic diagram is shown in Figure 9a. The test device consists of a transient pulse generator, a field formation device, and a power supply; the transient pulse generator is used to form an electromagnetic pulse. The field formation device simulates the formation of a 30 kV/m electromagnetic environment. The power supply provides power for the test circuit to keep the circuit in working condition.

## 5. Conclusions

Sensitive electronic components in a strong electromagnetic environment are easily interfered with or damaged. In this paper, a MERC is designed to channel the abnormal energy flowing through the pyrotechnic products. Through the combination of simulation, static experiment, and dynamic test, the working performance of the MERC is verified. The characterization test of the tested MERC is carried out to deeply analyze the effect of the MERC. The results show that:

(1) After the strong electromagnetic pulse injection, the pulse breakdown voltage of the MERC increases exponentially with the pulse injection voltage. The residual pulse current is significantly reduced from one-third to one-half of the original one, with an obvious protective effect. After comparison, the needle–needle structure of the MERC has a better releasing effect than other structures of the MERC.

(2) In the DC test, when the needle–needle structure of the MERC breaks down, there is a strong light, accompanied by an explosion sound, and the electrode metal melts while scorching occurs. The DC breakdown voltage for this design size is 144 V, and the on-time is around 0.5 ms.

(3) After the discharge breakdown of the MERC, the structure and morphology of the entire electrode is changed. The electrode gap increases significantly and the performance changes, making it unsuitable for repeated use.

Compared with traditional protection methods, MERC is small in size, in line with the trend of miniaturization of electronic products. Meanwhile, in terms of effect, it can effectively channel energy to sensitive electronic components and protect sensitive electronic components from electromagnetic environment interference. The future in microelectronics, automotive, weapons, and other fields has a broad application prospect.

## Figures and Tables

**Table 1:** Comparison of the advantages and disadvantages of various protection methods.

Component	Advantages	Disadvantages
Ferrite	effective absorption and attenuation of high-frequency-induced currents	no inhibitory ability to constant-current action, complex connection and package structure, difficulty processing
Zener diode	ns response speed, good voltage stabilization	high demand for breakdown field strength
TVS	ns response speed, absorbs 10^2^–10^5^ W pulse power	large junction capacitance, low breakdown voltage; not suitable for high-frequency environments
Thermistor	ns response time, μA leakage current, high-throughput	long response time and high temperature dependence
Gas discharge tube	µs-level response time, absorbs up to several kW of pulsed power	higher residual voltage and shorter life
Integrated circuits, filters	best protection, suppresses overcurrent to a low level	large device size, can only be used externally

**Table 2:** Simulation parameters of MERC.

Electrode Material	Electrode Width	Electrode Length	Tip Size
Au	50 μm	200 μm	Triangle, bottom edge 50 μm, height 50 μm
Au	50 μm	200 μm	Semicircle, radius 25 μm
Au	50 μm	200 μm	Square, side 50 μm

**Table 3:** Residual current versus actual current for different magnitude of injection current.

Target Pulse Current Injection Peak (A)	Actual Peak Pulse Current Injection (A)	Peak Residual Pulse Current (A)	Unit Pulse Current Rejection Ratio (dB)
50	47.6	24.4	5.8
100	106.2	32.6	10.3
200	194.2	59.6	10.3
300	291.0	132.0	6.9

**Table 4:** Element mass ratio before and after electrode surface.

Elemental	C	N	O	Si	Cr	Au
Pre-test (%)	3.16	0	0	2.71	0	94.14
Post-test (%)	6.65	6.97	0.80	45.80	3.92	35.86

**Table 5:** Mass ratio of each element on the electrode surface.

Number	C	N	O	Si	Cr	Au
A#1 (%)	6.89	13.66	1.00	69.94	1.45	7.05
A#2 (%)	9.69	10.83	1.07	67.12	1.60	9.68
A#3 (%)	4.31	1.34	0.39	17.79	6.08	70.10
A#4 (%)	5.73	2.06	0.72	28.35	6.55	56.59

## Data Availability

Datasets used within this study are available upon request.
